# Contextual Cluster-Based Glow-Worm Swarm Optimization (GSO) Coupled Wireless Sensor Networks for Smart Cities

**DOI:** 10.3390/s23146639

**Published:** 2023-07-24

**Authors:** P. S. Ramesh, P. Srivani, Miroslav Mahdal, Lingala Sivaranjani, Shafiqul Abidin, Shivakumar Kagi, Muniyandy Elangovan

**Affiliations:** 1 Department of Computer Science and Engineering, Vel Tech Rangarajan Dr. Sagunthala R & D Institute of Science and Technology, Chennai 600062, India; drpsramesh@veltech.edu.in; 2 Department of Computer Science and Engineering, BMS Institute of Technology and Management, Yelahanka, Bengaluru 560064, India; srivanicse@bmsit.in; 3 Department of Control Systems and Instrumentation, Faculty of Mechanical Engineering, VSB-Technical University of Ostrava, 17. Listopadu 2172/15, 70800 Ostrava, Czech Republic; 4 School of Computing, Mohan Babu University, Tirupati 517102, India; sivaranjani.l@mbu.asia; 5 Department of Computer Science, Aligarh Muslim University, Aligarh 202001, India; s.abidin.cs@amu.ac.in; 6 Department of Computer Science and Engineering, Sharnbasva University, Kalaburagi 585105, India; kagi@sharnbasvauniversity.edu.in; 7 Department of Biosciences, Saveetha School of Engineering, Saveetha Nagar, Thandalam 602105, India; muniyandy.e@gmail.com; 8 Department of R&D, Bond Marine Consultancy, London EC1V 2NX, UK

**Keywords:** multi-parameters, cluster head, retransmission ratio, glow-worm, optimization and heterogeneous

## Abstract

The cluster technique involves the creation of clusters and the selection of a cluster head (CH), which connects sensor nodes, known as cluster members (CM), to the CH. The CH receives data from the CM and collects data from sensor nodes, removing unnecessary data to conserve energy. It compresses the data and transmits them to base stations through multi-hop to reduce network load. Since CMs only communicate with their CH and have a limited range, they avoid redundant information. However, the CH’s routing, compression, and aggregation functions consume power quickly compared to other protocols, like TPGF, LQEAR, MPRM, and P-LQCLR. To address energy usage in wireless sensor networks (WSNs), heterogeneous high-power nodes (HPN) are used to balance energy consumption. CHs close to the base station require effective algorithms for improvement. The cluster-based glow-worm optimization technique utilizes random clustering, distributed cluster leader selection, and link-based routing. The cluster head routes data to the next group leader, balancing energy utilization in the WSN. This algorithm reduces energy consumption through multi-hop communication, cluster construction, and cluster head election. The glow-worm optimization technique allows for faster convergence and improved multi-parameter selection. By combining these methods, a new routing scheme is proposed to extend the network’s lifetime and balance energy in various environments. However, the proposed model consumes more energy than TPGF, and other protocols for packets with 0 or 1 retransmission count in a 260-node network. This is mainly due to the short INFO packets during the neighbor discovery period and the increased hop count of the proposed derived pathways. Herein, simulations are conducted to evaluate the technique’s throughput and energy efficiency.

## 1. Introduction

WSN contains many densely packed sensor nodes distributed across the monitored region. Low-cost nodes are able to communicate with one another over wireless links in order to convey their acquired data to the sink node. The result of the power limits of the nodes, however, is that energy saving becomes a significant design difficulty for WSNs. Also, as sensor nodes are frequently positioned in risky or difficult-to-reach areas, it may be difficult to charge up their cells. Hence, there is much research potential in the areas of network longevity and energy efficiency [1,2].

It has been demonstrated that interaction is the primary cause and measure of energy usage in WSNs [3]. In order to reduce energy usage, numerous routing methods currently in use were utilized to discover the shortest path for data transfer. Nevertheless, the shortest path strategy did not extend the lifespan of the network. Energy-efficient routing requires balanced network energy consumption [4,5].

WSNs with a single sink continue to pose several problems. Despite the fact that many academics have worked on the architecture in WSNs of routing schemes, there is only one sink that is commonly used to increase the network’s lifespan [6,7]. The main problem with multi-hop WSNs is that because they need to relay more traffic, sensor nodes near the sink node deteriorate more rapidly than those farther away [8,9]. The result is that the network’s longevity is unavoidably impacted by the power-hole problem caused by uneven energy consumption [10,11]. Thus, using WSN with a single sink might not be practicable [12]. Hence, multi-sink techniques have been devised for the purpose of preventing such issues [13,14,15], in order to circumvent or avoid the energy hole creation issue. To gather data from the sensing nodes, a number of sink nodes are positioned at preset locations throughout the monitored field. Furthermore, energy balancing between sensor nodes enables multi-sink architecture to effectively handle the energy-hole problem; this is superior to using a single sink in many ways. If there are multiple sinks, network throughput can be increased, and the data will still be transferred if one sink node fails for whatever reason.

The typical gap between sensor devices and sink nodes narrows as the utilization of many sinks decreases, along with data transfer latency and energy consumption. Last but not least, the network’s traffic congestion problem may be somewhat mitigated by the deployment of several sink nodes [16,17]. Nevertheless, as sensor nodes must decide which data transfer sink is optimal, the best way to select sink nodes is uncertain.

Another major obstacle is the fact that WSNs are frequently situated in adverse settings, which exposes sensor networks and node sinking to failure. Thus, the networks are rarely functional. For instance, in high temperatures, sensors and sink nodes become more susceptible to failure and, perhaps, total destruction. Additionally, their probability of short-circuiting is increased by the exceptionally high humidity [18]. Thus, it is crucial to mitigate factors affecting network performance, like humidity and temperature. Hence, transmission of data should be stopped in hazardous regions (for instance, regions that are situated in severe environments). Data delivery should be delayed whenever the data flow through dangerous area, like a fire zone, as a result of relay nodes that have been destroyed. Hence, environmental considerations should be factored into the algorithm design.

WSNs may provide a solution for many forms of real-time software, including manufacturing automation, but sensitive information must be transmitted in real time to the sink node (deadline). In this circumstance, accurate data exchange ensures that the proper measures are taken, but late delivery of data compromises the success of this action. Thus, when creating the routes, this problem must be considered.

Another difficult aspect of WSN is the dependability of the transfer of data, or the assurance that the data will reach their destination. Owing to the intrinsic nature of wireless communication in sensor networks, packet loss is unavoidable. Due to environmental factors, such as fading and interference, wireless links are susceptible to network interruptions. This increases the likelihood of retransmission for missing packets, resulting in increased energy usage and delivery delays. Consequently, while creating the routing strategy for WSNs, it is also necessary to consider the quality of wireless links.

As a result of its adaptability and versatility in handling several complicated problems, (SI) methodologies are created to provide efficient optimization methods for a variety of WSN scenarios in order to address the aforementioned issues. Ant colonies, bee swarms, and animal swarms all have an influence on swarm intelligence optimization strategies [19,20,21]. Social ant colonies are similar to the (ACO) strategy. The quality of the solution can be determined by looking at the pheromone concentration in ACO. Usually, the problem is brought on by pheromone levels [22,23].

There have been two work periods for this investigation. Choosing the best sink was the first step. While many academics have concentrated on challenges related to optimal sink selection, an effective sink node selection strategy was put forth in the first phase. The majority of studies attempted to choose the best option on the basis of node power and transport power usage [24,25] without taking into account environmental influences, which can be changed by path modifications. In difficult situations, sink node selection is unexpected. In order to reduce the sink’s negative effects on the environment, this article applies environmental awareness [26,27,28]. Second, the speed and/or delay of the real-time flow of data is considerably impacted by delivery delays of real-time data packets. Lastly, it is essential to weigh the costs of power against the benefits of attaining the timing of delivery while selecting the optimal sink algorithm to reduce the packet miss percentage (the proportion of delivery packages that were late) [29,30]. In order to enhance the network’s lifespan by equalizing the power usage of all sink nodes, the sink load metric is displayed last.

According to our knowledge, the routing algorithm makes up the next stage; various research publications have investigated the multi-sink WSN routing issue. The vast majority of proposed solutions strive to improve energy efficiency [31,32]. The ecological impact of routing protocol architecture is excluded from such studies. As a result, they are unable to swiftly adjust to environment stresses such as wildfires and storms. To the best of our knowledge, only one routing technique increased routing dependability and energy effectiveness in hostile settings at the time this work was being produced. This approach also considered environmental awareness. The drawback of this method is that it cannot be used in real time. In an effort to lessen the detrimental effects of the environment on data transmission and make real-time applications appropriate, this paper presents a routing strategy. It also assesses the network’s quality in terms of preventing the transmission of data packets through faulty routes. In order to balance energy usage, a unique function linking lingering power with the sensor network load is recommended. The following are the most important contributions:

- Offering environmentally conscious, energy-efficient real-time routing for multi-sink WSNs that takes into account environmental information, link strength, load balancing, energy, and latency;
- Describing the issue with routing by means of a large-scale WSN with several sinks using 0/1 numerical programming;
- The issue with the route is expressed as 0/1 integer programming so that it can be utilized by other researchers;
- Leveraging the emergent intelligence of a swarm with the aim to enhance the routing process, optimize network performance, and improve the overall efficiency of the WSN—as a heuristic outcome, swarm intelligence is utilized.

Henceforth, the primary highlight of this work is aligned with the goal of prolonging the network’s lifetime. By developing an environmentally conscious and energy-efficient routing approach, considering factors such as load balancing, energy consumption, and environmental information, the aim is to optimize energy usage and extend the overall lifespan of the network. Additionally, the utilization of swarm intelligence as a heuristic approach can contribute to the efficient utilization of network resources and further enhance energy efficiency. An outline of the paper is provided below. The most relevant work is described in Part 2, while the issue that needs to be resolved is mentioned in Section 3. The issue is defined in Section 4. The implications are described in depth in Section 5. Section 6 contains the conclusion of the paper.

## 2. Related Works

4G LTE and (LTE/A) use Internet Protocol-based packet switching (IP). The fundamental challenge of LTE is accommodating fluctuating customers within a given area. Using (ML)-based many input many output, as well as channel prediction based on user resource acquisition, network fluctuation is achieved (MIMO). In order to increase the precision of CSI with delayed processing and compression of data [33], channel rate adaptation [34] is used for imperfect CSI. This strategy, however, is ineffective when used with 5G networks due to the immense volume of traffic. The authors of the study [35] suggested distributing resources in accordance with an algorithm that pairs end users with their respective RRH. The approximate method calculated how many end users were linked to a certain RRH. The client and RRH, as well as RRH and BBU, then became connected. The resource allocation system presented by the authors in [36] integrates a LTE network and new radio to centrally address the issue of complicated calculations and overhead signals. The proposed HCCRRA improved throughput and power usage according to the fluctuating user pace of packet arrival. The distribution method, which is dependent on various timelines and congestion control recommendations, can be employed in the upcoming 5G networks [37].

The study referenced in [38] examined a time-delay pool created for hybrid beamforming and the challenge of millimeter-wave (mm wave) beamforming (for cases with a single user and numerous users in 5G CRAN networks). By analyzing network traffic in relation to resource demand in the CRAN network, an energy-efficient method based on resource provisioning was developed [39]. These researcher considered the issue of controlling and optimizing the resource while using a strategy for resource supply. The resource allocation was created by the authors using GCNs [40]. They showed that the suggested technique greatly lowers the wireless networks’ communication complexity [41]. The process is more energy-efficient, since more resources are used when the network is denser. The cooperative approach, which employs the recommended allocation of resources based on the model of filling with water boost power consumption based on the end-characteristics, is described in [23] to merge transmission and power consumption.

Similarly to this, the authors of [42] suggested a resource allocation method that would verify the output of the binary classifier using a system scheduler and the random forest algorithm. Although the approach operated well in terms of robustness, there was little room for further research and development. The random forest algorithm was recommended by the authors of [43] as a method for creating a classifier using supervised machine learning. The authors considered categorizing the input parameters which were utilized using the ID3 decision tree. In addition, the SINR was maintained and kept constant when distributing resources. Nevertheless, because the SINR value is dynamic, we might not be able to determine the best way to allocate resources.

After researching several methods of source distribution among BBU in CRAN, we found that like (HPN) tier, RRH tier, and HCRAN, among others, the typical method of RA in fifth-generation (5G) networks utilizes CSI-based end user (EU) information. The typical RA technique is not suitable for implementation in CRAN for a 5G network [44] due to its massively higher system complexity (about 25% of the entire capacity of the system).

When the system’s overall user base grows, the old CSI-based RA approach likewise fails to deliver optimal outcomes. In addition, the decision tree used to forecast MCS in machine learning-based RA schemes is ID3 when test or learning data have null values, which is useless. Moreover, the solution for overfitting is not addressed by ID3 decision trees. The data pre-processing and categorization techniques used in machine learning increase the precision of the prediction process [45]. Scheduling framework-based resource computation opens the door for data-reliable resource management [46]. Successful resource segmentation is proposed in [47], which explains the benefits of CRAN’s resource allocation effectiveness management system. Hence, the proposed method allowed for the application of the current random forest algorithm to RRH with MCS schemes. Analysis of the average throughput over user count and scatter density was conducted.

### Research Gap Identified

To overcome the aforementioned difficulties, the author advises employing the (EU) position estimates. The machine learning-based supervised RA approach which we propose can use the EU’s position estimates as an input with which to produce a C4.5 decision tree that can distribute MCS to the EU [48]. The authors thus seized this chance to suggest an effective RA scheme for integration into the CRAN system for the 5G network. As a result, the goal of our suggested technique was to allocate resources by effectively using a decision tree and a modified random forest algorithm [49,50]. The objectives of the proposed work are:

- To develop an environmentally conscious and energy-efficient real-time routing approach for multi-sink wireless sensor networks (WSNs) that considers environmental information, link strength, load balancing, energy consumption, and latency. The aim is to optimize routing decisions in order to minimize energy usage and enhance the overall efficiency of the network.
- To investigate the challenges associated with routing in large-scale WSNs with multiple sinks. Specifically, we employ 0/1 numerical programming to express the routing problem as a binary decision problem, allowing for precise formulation and analysis of the routing issue.
- To express the routing problem as 0/1 integer programming to facilitate its utilization by other researchers. By converting the routing issue into a well-defined binary programming framework, it becomes more accessible for further study, analysis, and the development of novel routing algorithms by the research community.
- To utilize swarm intelligence as a heuristic approach to address the routing challenges in multi-sink WSNs, as well as to leverage the emergent intelligence of a swarm, aim to enhance the routing process, optimize network performance, and improve the overall efficiency of the WSN.

Hence, these technical objectives highlight our aim of developing an energy-efficient routing mechanism, investigating and formulating the routing issue for analysis and utilization, and utilizing swarm intelligence as a heuristic technique with which to tackle the identified challenges.

## 3. Proposed Methodology: Contextual Clustering

**Figure 1:**
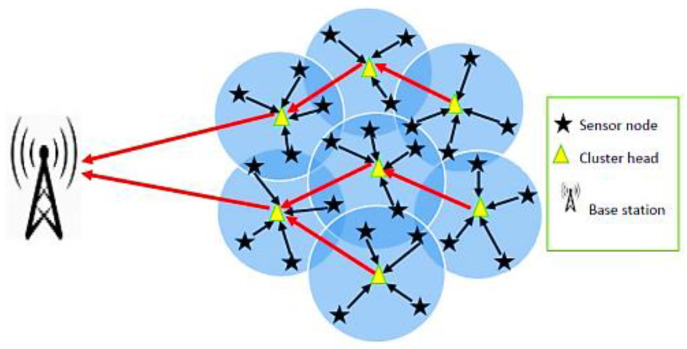
Cluster-based distributed WSN.

The primary objective of this work is to create an algorithm that clusters and routes information in a way that balances the load and prolongs the lifespan of a WSN. We propose a protocol called cluster-based energy-resource-efficient routing protocol (CBERERP), which is founded on the heterogeneity of sensor nodes. The communication between various clusters and the base station in the deployment area is depicted in Figure 1.

The cluster-based CBERERP is composed of various sensor nodes that differ in terms of storage, energy, and computation capacity. Nodes with high energy are referred to as HPN. These heterogeneous nodes aid in balancing energy consumption and increasing the network’s lifespan. Nodes with heterogeneous characteristics have a greater chance of being chosen as cluster heads, which facilitates longer transmission in the WSN. The CBERERP algorithm follows a three-step approach, including the selection of cluster heads, cluster formation, and inter-cluster routing. The algorithm’s complete mechanism can be summarized as follows (FIFO).

In a distributed environment, the choice of CH involves the participation of every sensor node within its range in the cluster head selection algorithm. The node with the most power is selected as the CH. The cluster-based power-resource-efficient routing protocol (CBERERP) operates on a distributed concept, where independent heterogeneous nodes use a message-passing model to communicate. Initially, the nodes are unsynchronized and unaware of their locations. The CBERERP algorithm proposes that ∀i: 1 ≤ i < N ≤, where N, Si, and Ri represent the total number of sensor nodes, unique sensor nodes, and unique request sets, respectively, and Ri = S1, S2, …, Sn. Each unique sensor node has a unique request queue that stores requests sent by other sensor nodes and orders them based on their time stamp values. The time stamp value is assigned to every request according to the available energy of the node and is represented by an integer number, which is assigned by the algorithm and defined in the equations given below. Sensor nodes process requests from the queue using the first input first output order. The CH selection in CBERERP is completed in three phases, including participation of the sensors in CH election, fulfillment of the conditions to become CH, and exit after task completion. The three steps of the CH selection algorithm are given below:

(a) Request phase for CH selection.

If a sensor node $S_{i}$ desires to be a cluster head (CH), it will broadcast a REQUEST (tsi,i) message to all other sensors listed in Ri and keep a record of this request in the request-queue i(tsi,i), with (tsi,i) representing the timestamp of the given request i. If another sensor node Sj also wants to be a CH and receives the REQUEST (tsi,i) message from Si, Sj will reply to Si with a REPLY message that contains the timestamp value. Sj will store Si’s request information in its request queue j.

(b) Conditions phase to become a CH.

Once a sensor node successfully completes the first phase, it becomes eligible to become a CH. However, there are two necessary conditions that must be met for the selection of a CH, and a sensor node Si is only selected as a CH when these conditions are satisfied. The two necessary conditions are as follows:

Condition 1: The Si sensor node receives requests from other sensor nodes interested in becoming a CH. It will only become a CH if it receives requests from other sensor nodes with time-stamps larger than its own time-stamp (tsi,i). A lower time-stamp indicates higher energy availability, while a higher integer number indicates lower available energy. This condition ensures that only sensor nodes with high available energy are selected as CHs.

**Figure 2:**
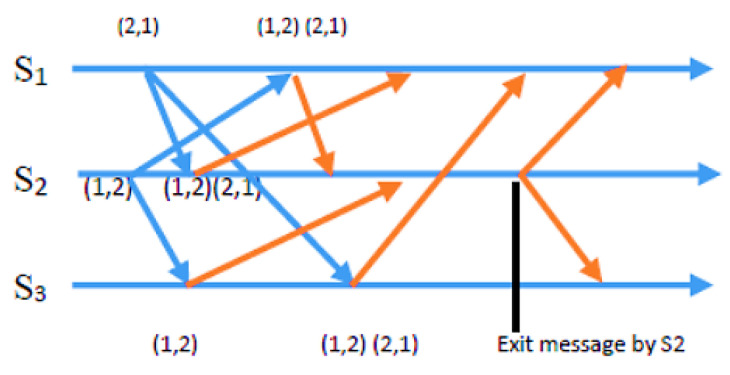
Exit info from S2.

**Figure 3:**
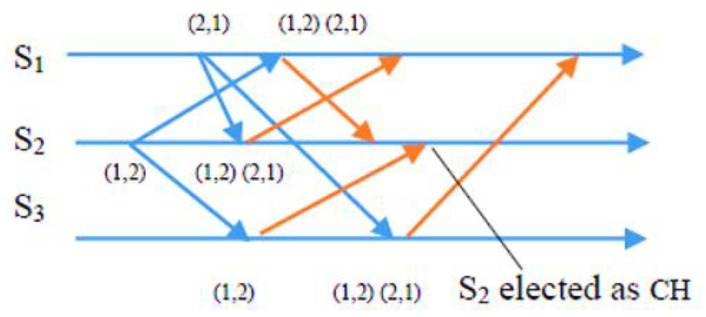
Sensor S2 elected as CH.

**Figure 4:**
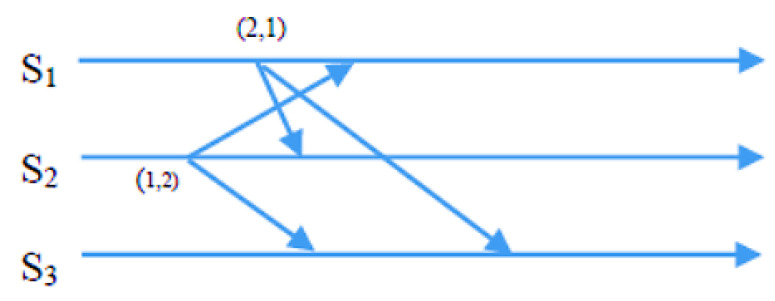
Sensor S2 broadcast release message.

**Figure 5:**
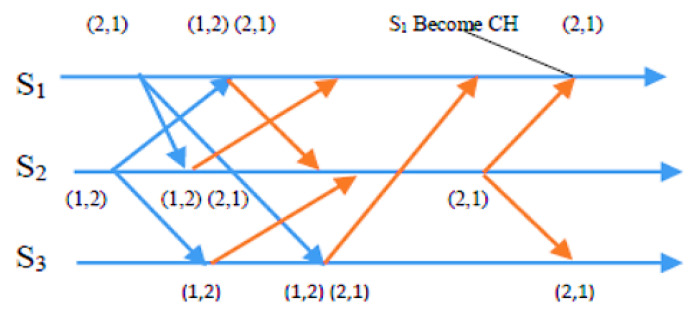
S1 becomes CH in the second set of sensors.

Condition 2: To ensure fairness, the Si requests from sensor nodes must be at the top of the node’s own request queue i, which follows a first-in, first-out (FIFO) order. In the exit phase of the algorithm, a sensor node Si gives up its CH role once its energy level drops below a specific threshold. This enables nodes with higher energy levels to assume responsibility and balance the load, extending the network’s lifespan. Si achieves this by removing something from the front of its request queue and transmitting a time-stamped RELEASE message to all other sensors in its requests. When other sensor nodes receive the RELEASE message, they delete Si’s entry from their request queues. This can lead to Sj’s request being at the top of the queue, allowing Sj to take over as the new CH. The process of the request, condition, and exit phases is demonstrated in detail through CBERERP in Figure 2, Figure 3, Figure 4 and Figure 5. To explain the process thoroughly, three heterogenous sensor nodes are considered in Figure 5. Sensor nodes S1 and S2 express their interest in becoming a CH by requesting, while sensor node S3 does not participate due to either low energy availability or having already played the role of CH.

In a scenario with *n* = 3 sensor nodes, S1 and S2 send REQUEST messages to the other two nodes. The distributed algorithm utilizes each node’s available energy to assign timestamps. S1’s REQUEST message has a timestamp of (2, 1), while S2’s REQUEST message has a timestamp of (1, 2). After broadcasting the REQUEST message, sensor nodes remain idle and wait for REPLY messages from *n*-1 other sensor nodes (in this example, *n* = 3). As depicted in Figure 3, S2 received REPLY messages from S1 and S3. Since S2 met the necessary requirements and was at the top of its request queue, it became the CH for this round. The other sensor nodes continued to wait until S2 hit the energy threshold and transmitted a RELEASE message to relinquish its role.

As depicted in Figure 4, when S2’s energy level reached the threshold point and it desired to relinquish the CH role, it transmitted a RELEASE message to *n*-1 other sensors (where *n* = 3). S1 and S3 received this message. Concurrently, S1 received REPLY messages from S2 and S3, and it is now at the top of its queue. Consequently, S1 is qualified to become a CH in the second round, as demonstrated in Figure 5, which illustrates the second round of the algorithm.

The time-stamp value in this algorithm is determined by each node based on its available energy. The value is an integer, with a maximum cluster size of 100 nodes. The time-stamp is assigned in reverse order of the node’s total available energy percentage. For instance, a node with 100% energy is assigned a time-stamp value of 1, while a node with 99% energy is assigned a value of 2, and so on. The time-stamp values follow an inverse relationship with the available energy. The CH is selected by the algorithm for a maximum period of T_CH, after which the node may elect itself as the CH and broadcast the announcement to the cluster.

### Developing Clusters in a Distributed Environment

**Figure 6:**
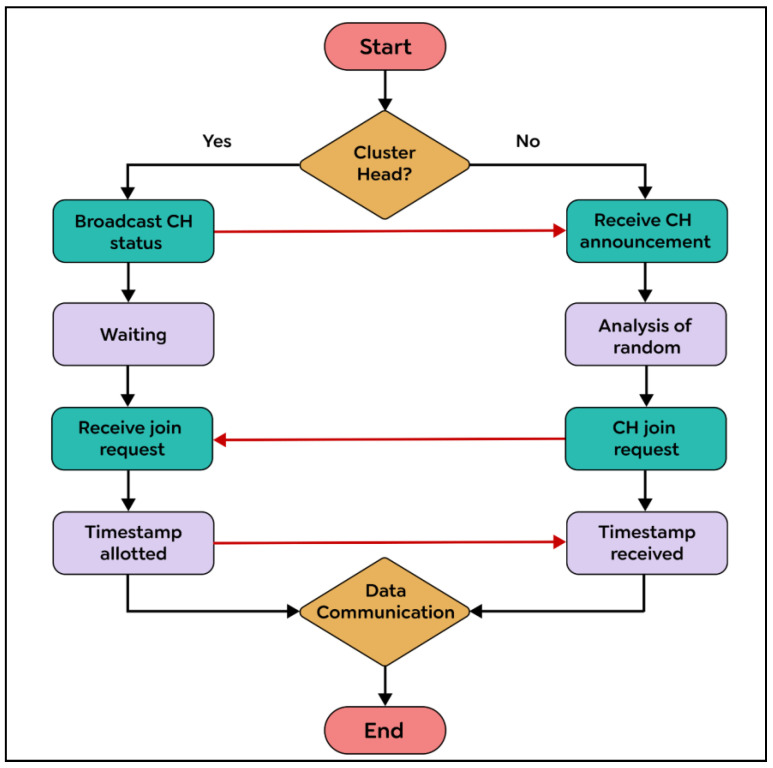
Cluster development for non-CH.

During the cluster formation phase, the CH selects nodes within its radius to form a cluster. The cluster formation and CH election tasks are both performed using the CBERERP algorithm, eliminating the need for a separate algorithm. The algorithm first uses the request condition exit (RCE) distributed technique to elect the CH. Once elected, the CH has all the information about the nodes within its range, including their locations, available energy, and identification numbers. Each node maintains a request queue, which stores information about all other nodes, excluding those not participating in the CH election process, already elected as a CH, or low on energy. The CH forms a cluster with these known nodes, while the nodes not included in the CH election process or with low energy join the nearest CH within their range. When a node comes in the range of multiple CHs, it uses a selection process called the random method to choose the low-energy CH closest to it. If that CH is not available, the node selects the CH with the longest range and highest energy to distribute the load and improve the network’s lifetime. The process of cluster formation and CH selection is explained in detail in Figure 6. Once elected as a CH, node i can initiate the CBERERP algorithm by broadcasting an announcement message to establish a cluster. Other sensor nodes can then join the cluster, while those not participating receive the announcement and use the priority-based random algorithm to select their CHs. The non-CH node k sends a joining REQUEST message to the identified CH with its time-stamp value. The CH accepts the request, synchronizes the sensor node based on the time-stamp value, and forms the cluster. Once formed, the inter-cluster and intra-cluster routing algorithm is activated to identify the path for data exchange between the sender and receiver nodes. In this algorithm, CH plays a critical role in routing. Additionally, the proposed CBERERP algorithm includes a balanced routing mechanism based on next hop information.

The proposed energy-effective knowledge-based routing for the next jump used three phases: atom setup, accuracy function, and topology update. We suggested a next-hop-based routing algorithm. The actions are described below.

1. Atoms Initialization Phase (A Unique Next Hop Selection):

Each cluster head is associated with a unique route to the control room, and this path is defined by a set of atoms. To the nearest integer, the number of atoms is proportional to cluster heads in the deployment area. An atom is represented as Xi,k, where 1 ≤ i ≤ N and 1 ≤ k ≤ M. Here, N is a predefined function that represents the size of the network, and M is the total number of cluster heads. The value of Xi,k indicates the next cluster head in the route to reach the base station. It is generated randomly, such that 0 ≤ Xi,k ≤ 1, and it identifies the next cluster head CHp from the current cluster head CHk. The formula for the connection between CHp and CHk is expressed as follows: CHp = F(R_next_hops(CHk),*n*), where F is an indexing function, CHk is the existing cluster head, CHp is the next cluster head along the path, and *n* represents the ceiling function of (Xi,k × |R_next_hops(CHk)|).

**Figure 7:**
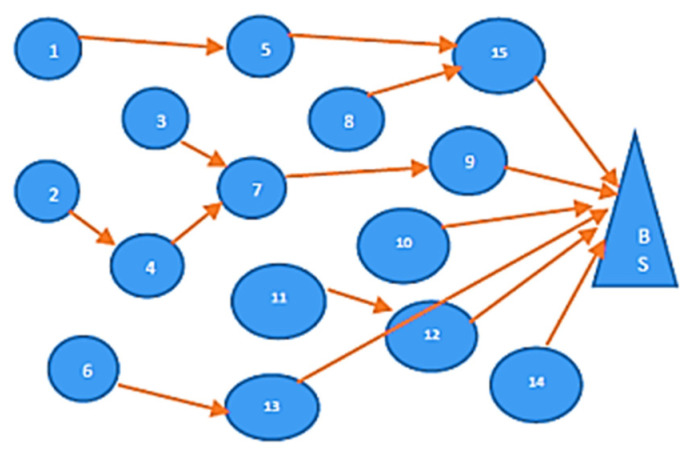
15 CH in WSN.

To illustrate this, Figure 7 shows a set of 15 cluster heads (CH1, CH2, …, CH15), and the number of atoms M in this example is also 15, which is determined during the atoms’ initialization phase. The cluster heads in the example form a directed and acyclic graph, where the edges represent the path towards the BS between the cluster heads. If there is an edge from CHi to CHk, it indicates that there is a path from CHi to CHk, and that CHk is located within the range of CHi, closer to the BS. For instance, based on Figure 7, CH2 has three next hops to reach the BS (via CH3, CH4, and CH6) or R_next_hops(CH2) = CH3, CH4, CH6. The K-NH routing displays the next hop of all the cluster heads towards the BS, which is determined using the previously defined function.

In the proposed algorithm, a random quantity between 0 to 1 is given to each CH based on its available energy, where 100% energy is represented by 1, 99% energy is represented by 0.99, and so on. For each atom Ai, which represents the mean of the available energy of all possible next hop (R_next_hops(CHi)), a value Xi,k between 1 and 15 is assigned. The node’s available energy is given by [0.7, 0.8, 0.7, 0.8, 0.5, 0.5, 0.7, 0.5, 0.6, 0.8, 0.9, 0.7, 0.6, 0.7, 0.6] for [CH 1–15], respectively. Each atom Ai provides a complete route from each CH to reach the BS. For instance, consider Xi,2 = 0.67 for CH2, which is the mean of the available energy of nodes CH3, CH4, and CH6. Using the CHs, R_next_hops(CH2) = CH3, CH4, CH6, which are given in column 2, and the vector according to the ceiling (Xi,2 × |R_next_hops(CHk)|) is 2, as shown in column 5. This value indicates that CH2 chooses CH4 out of three existing paths to reach the BS. By applying the proposed algorithm to the directed and acyclic graph shown in Figure 7. The selection of the CH can be performed through distributed algorithms such as cluster-based glow-worm optimization, where each sensor node participates in the process. Various criteria have been considered, such as energy level, distance to the base station, and a combination of factors like residual energy and communication cost. These measures typically involve a probabilistic approach, where nodes with higher energy levels have a higher chance of becoming CHs. The selection process can progress in rounds or stages to ensure fair distribution of the CH role among the nodes. To balance energy consumption and prolong the network’s lifetime, the proposed work facilitates rotation of the role of CH among the nodes. This helps to prevent individual nodes from depleting their energy more quickly than others. The rotation frequency depends on the network’s characteristics and requirements. It can be predetermined based on the network’s lifetime or adaptively determined by monitoring energy levels and dynamically adjusting the CH selection probabilities.

2. Accuracy Function Phase

The goal of our proposed effort is to reduce communication distance and the hop count, i.e., the complete number of relays of the BS. The aim is to reduce the communication distance, which is represented by Mc and calculated as the minimal separation of CHi and its next hop, i.e., Min (dis (CHi, NextHop(CHi))), where i ranges from 1 to M. The objective is to minimize the number of hops, with Mh denoting the minimum number of hops needed to reach the base station for every cluster head CHi, where 1 ≤ i ≤ M. The two presented goals are at odds with each other, as decreasing the communication distance between nodes results in an increase in the number of hops required, and vice versa. This poses an NP complete problem, but the next hop knowledge routing algorithm can provide a solution that balances these objectives [51,52,53]. To achieve a multi-objective solution, a random weight is multiplied with each objective and the products are summed to obtain a single scalar objective as follows: Accuracy = W1 × Mc + W2 × Mh, where W2 = 1 − W1 and 0 ≤ W1 ≤ 1. The algorithm’s aim is to minimize the accuracy function, which corresponds to selecting a suitable atom Ai.

3. Topology Update Phase

When there is a change in the graph of a node, the topology is updated. The proposed method utilizes the concept of sleep nodes, active nodes, and node failure, which can result in changes in the network’s topology. These changes also affect the values of atoms, and frequent changes can make them unstable. Therefore, after any topology change, the accuracy function evaluates the atom Ai. This mechanism is known as topology fitness (Tfit), and it is self-changing. The updating process is as follows:

Abest_i_ = A_i_ if (accuracy (A_i_) < accuracy (Abest_i_)) otherwise Abest_i_ (1)

**Figure 8:**
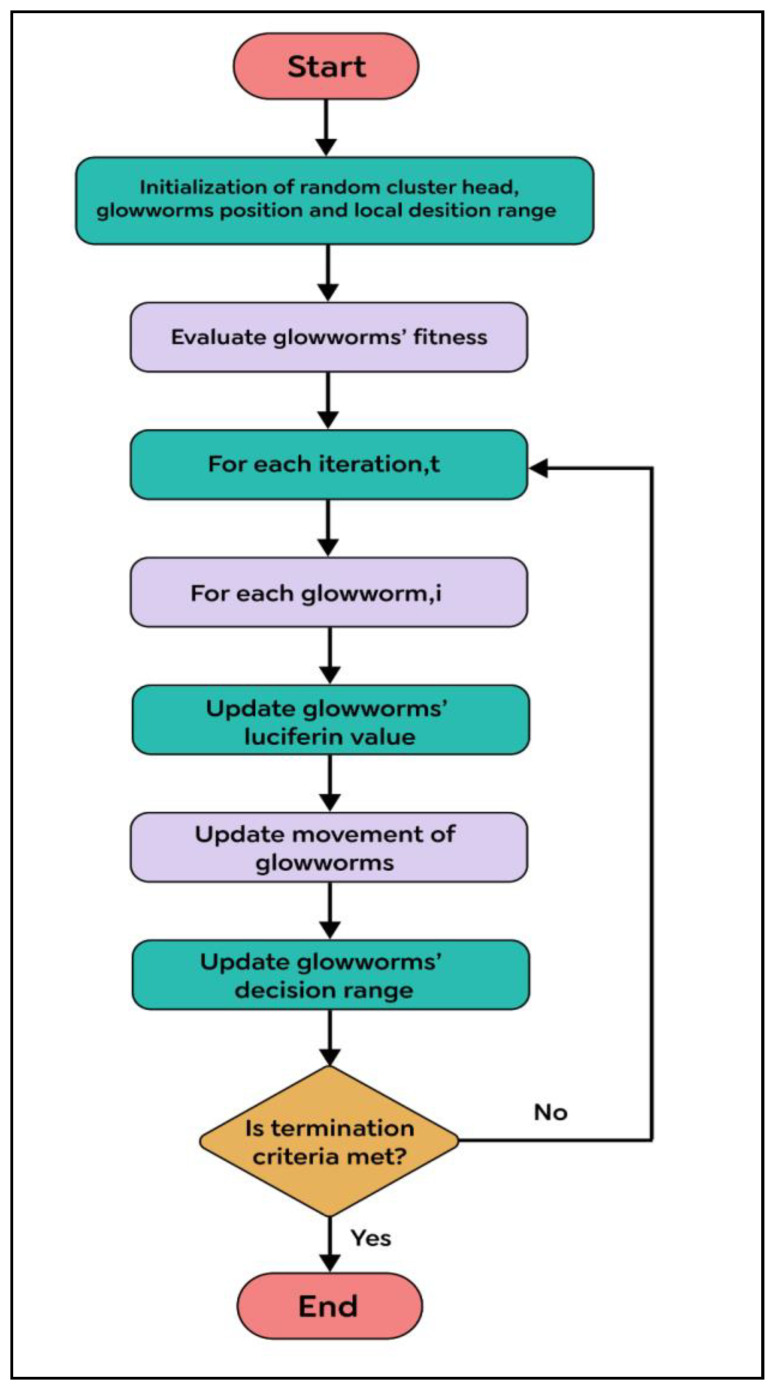
Flowchart for the GSO algorithm.

The proposed algorithm aims to achieve optimal outcomes by choosing CH2 as the next hop in the first round for CH1, but in the subsequent rounds, CH1 selects a different CH in order to spread the load and increase the network’s lifespan. The ultimate path to the base station is determined by Abest_i_, which is revised in each iteration to ensure load balancing. Figure 8 shows the pseudocode for the algorithm. The K-NH Routing is shown in Table 1.

The article discusses the utilization of the GSO algorithm for optimizing a WSN approach to localization. By using the GSO algorithm to select optimal parameters, it becomes possible to avoid adjusting parameters in EEHC. The optimization process involves the m0, α, and β parameters found in the EEHC. The GSO algorithm employs agents that scan the search space simultaneously, seeking fitness in their current positions. The agents determine fitness based on the level, which indicates the amount of glowing. Once the agent identifies a randomly chosen neighboring position, it moves and broadcasts a higher value of luciferin. If the agent does not have any other neighbors, it can become stuck without moving, which is a problematic characteristic that can lead to load imbalance in parallel processing.

The location of the glow-worm is determined when I t is denoted by xi (t) during the Luciferin-update stage, and the exact primary purpose there is J (xi (t)). Equation (2) is used to calculate the glow-worm i’s luciferin level at time t.

(2) $$l_{i}(t)=(1-\rho )l_{i}(t-1)+\gamma J\left(x_{i}(t)\right)$$

The given equation involves two variables related to luciferin, namely, the luciferin decay constant, represented by ρ (0 < ρ < 1), and the consistent luciferin improvement, represented by γ. During the movement phase, the glow-worm algorithm employs Equation (3) to determine the value of Ni (t), which represents the identification of a neighbor j for each glow-worm i.

(3) $$j\in N_{i}\;\mathrm{iff}\;\mathrm{Dis}\;\tan ce_{ij}<rd_{i}(t)\;\mathrm{and}\;l_{j}(t)>l_{i}(t)$$

The movement of glow-worm i towards its neighbor j is determined by a probability calculated using Equation (4).

(4) $$\begin{array}{c} \begin{array}{cc} & p(t)=l_{j}(t)-l_{i}(t)\\ & \;\mathrm{ij}\;\sum l(t)-l(t) \end{array}\\ k\in N_{i}(t)N^{k} \end{array}$$

To update the position of the glow-worm i, Equation (5) is used.

(5) $$rd_{i}(t)=min\left\{rs,max\left[0,rd_{i}(t-1)+\beta \left(nt-|N_{i}(t-1)\right)\right]\right\}$$

The local-decision range, which represents the neighborhood, is updated using Equation (6), with st denoting the step size.

(6) $$x_{i}(t+1)=x_{i}(t)+st*\left\{\frac{x_{j}(t)-x_{i}(t)}{∥x_{j}(t)-x_{i}(t)∥}|\right.$$

The GSO flowchart, depicted in Figure 8, includes two parameters: β, which controls the number of neighbors, and nt, which is a constant parameter.

The GSO algorithm comprises several steps:

- The initial phase involves the random initialization of a cluster head, the glow-worms’ position, and local decision range (Step 1);
- The fitness function of the glow-worms is evaluated (Step 2);
- During each iteration (Step 3), for each glow-worm (Step 4), if the number of glow-worms is too small, the glow-worm increases its local decision range to find more glow-worms (Step 5);
- Next, the luciferin value of the glow-worms is updated using the luciferin update rule (Step 6), and their movement is updated through a probabilistic mechanism (Step 7);
- The decision range of the glow-worms uses the neighborhood range to update the rule (Step 8);
- If the termination criteria are met, the algorithm ends (Step 9); otherwise, the process returns to Step 3.

## 4. Empirical Setup and Analysis

In this section, the proposed protocol’s multi-path performance is compared to that of other protocols in the absence of information service differentiation, as well as without frame retransmissions. PROPOSED is compared to TPGF, LQEAR, EGMFT, MPRM, and P-LQCLR utilizing routing protocols in the NS-2.35 network simulator.

Within a sensing area of 200 m × 200 m, all sensor nodes are stationary and scattered randomly. The source of the area is at (25, 100), and the sink node is at (175, 100). A single CBR data flow is generated at 64 kbps, then sent between the source and the sink over two different paths. Based on the triangle metric, a window of 50 HELLO packets is used to measure the quality of an asymmetric link. To maximize the QoS performance, weighted constants are set to 0:6, 0:2, and 0:2 in the PROPOSED, LQEAR, and P-LQCLR protocols. Table 2 provides a list of the key simulation variables and values that were applied to the simulations. One of two scenarios is used to assess the performance of each of the six treatments. The first scenario assesses the MAC sublayer frame retransmission-free routing performance for networks with 160, 180, 200, and 220 nodes, whereas the second scenario assesses routing efficiency using MAC sublayer frame re-transmission for networks with 160 nodes. Performance evaluations of both methods are based on in-depth simulations that are run individually for each situation in this section.

The evaluation of the six protocols includes assessing their performance, with and without MAC sublayer frame retransmission, in a network with 260 nodes. The performance metrics used to evaluate PROPOSED-P and compare it with the other protocols are jitter, PDR, energy consumption, and end-to-end delay.

### 4.1. Scenario with No Frame Retransmission

When a multimedia data transmission failure occurs in this case, neither the source node nor the intermediate nodes retransmit the data frame; this stops the MAC sublayer of IEEE 802.15.4 from sending frames again. Using random seed values, a total of 40 random network topologies are generated for networks with 160, 180, 200, and 220 nodes. For each network size of 160, 180, 200, and 220 nodes, the results of numerous simulations are averaged independently. PDR, end-to-end delay, jitter, power usage, and the network’s lifespan are used in the evaluation as network performance characteristics. Network lifetime is a performance metric that measures the duration for which a wireless sensor network (WSN) can operate effectively before the energy of the network nodes depletes to a point where they can no longer perform their tasks. In WSNs, sensor nodes are typically powered by limited energy sources, such as batteries. The network lifetime is defined as the time duration from the network’s initialization until the first node depletes its energy or reaches a critically low energy level, rendering it unable to function properly. Maximizing the network lifetime is desirable in WSNs, as it contributes to prolonged operation and increased monitoring capabilities.

Average packet loss per day as a result of interference: This performance metric assesses the impact of interference on packet loss in a wireless sensor network over a daily time frame. It quantifies the average number or percentage of data packets that are lost or not successfully received at their intended destination due to interference events occurring during the day. Interference can result from various sources, such as overlapping transmissions, noise, or external devices operating on the same frequency band. The average packet loss per day due to interference reflects the reliability and robustness of the network’s communication in the presence of interference, indicating the effectiveness of measures taken to mitigate or manage interference.

To calculate the average packet loss per day as a result of interference, we used the following formula:

Average packet loss per day = (total number of lost packets due to interference)/(total number of packets transmitted within a day). This metric provides an estimation of the impact of interference events on packet delivery and overall network performance.

**Figure 9:**
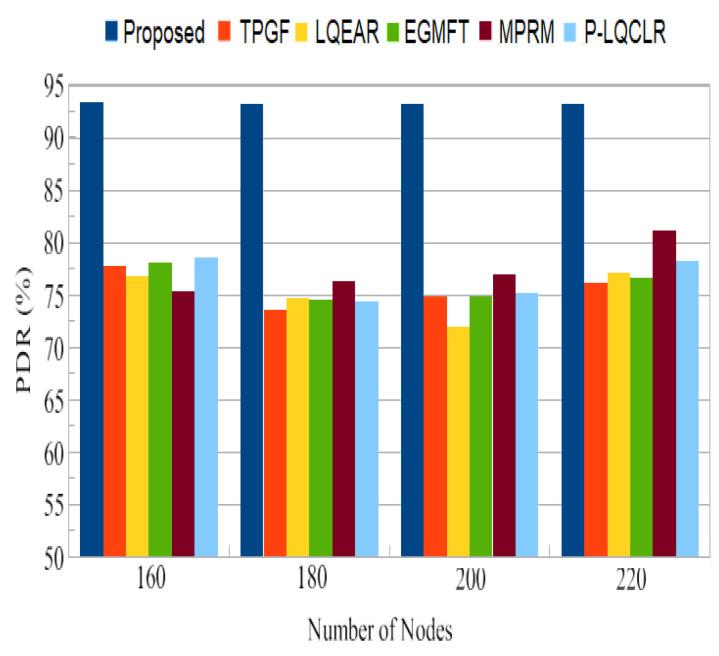
Ratio of average packet delivery.

**Figure 10:**
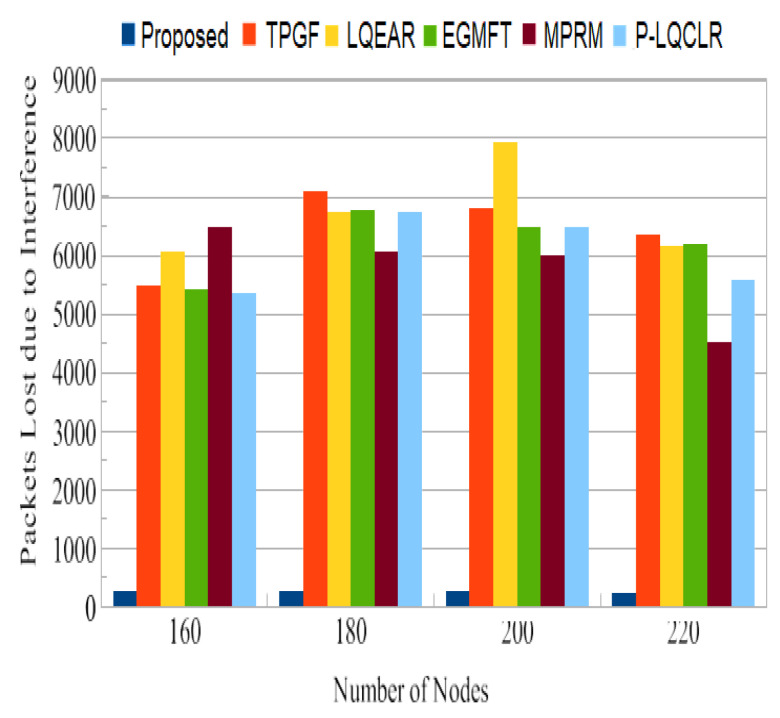
Average packets lost per day as a result of interference.

The primary network performance metric used to assess the reliability of the routing system in this study was the packet delivery ratio (PDR). PDR measures the proportion of packets successfully delivered to the sink node compared to the total packets generated at the source node. In Figure 9, the typical PDR values for the PROPOSED protocol and other protocols are shown. It was noted that the PROPOSED protocol achieved an average PDR close to 93%, while the other protocols ranged from 73% to 81%. This indicates that the PROPOSED protocol outperformed the other protocols in terms of successful packet delivery. Furthermore, Figure 10 illustrates the overall quantity of information packet losses caused by interference from neighboring nodes. It can be observed that, compared to the PROPOSED protocol, TPGF and the other protocols experienced a significantly higher number of data packet losses. This can be attributed to the strong inter-path interference impact discovered by TPGF and other protocols, as well as the presence of HNP (heterogeneous high-power nodes) at the sink node. The competition for channel access among close paths leads to a higher probability of packet collisions and increased packet loss. In contrast, the PROPOSED protocol identifies paths that have minimal adjacent path interference, resulting in fewer packet losses.

**Figure 11:**
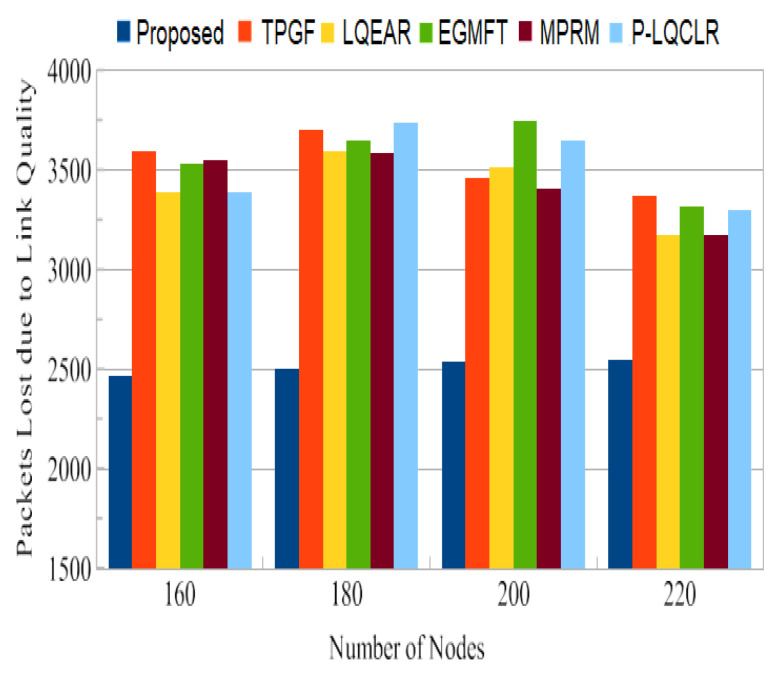
Average packets lost by physical layer as a result of poor connection quality.

Figure 11 presents the total amount of data packet losses caused by poor link quality. It can be observed that the other protocols experienced 27% to 31% more packet losses due to poor link quality than the PROPOSED protocol. The PROPOSED protocol demonstrated lower packet loss due to link quality because it selected a higher percentage of good-quality links; this is estimated based on the measure of triangle links’ quality. The lower PDR values observed in the other protocols can be attributed to factors such as inter-path interference effects, HNP at the sink node, and a higher percentage of poor-quality links on active paths. In summary, the results indicate that the PROPOSED protocol can achieve a higher PDR and experiences fewer data packet losses due to interference or poor link quality compared to the other protocols evaluated in the study. These findings highlight the superior performance of the PROPOSED protocol in terms of reliable packet delivery and mitigating the effects of interference and link quality issues.

**Figure 12:**
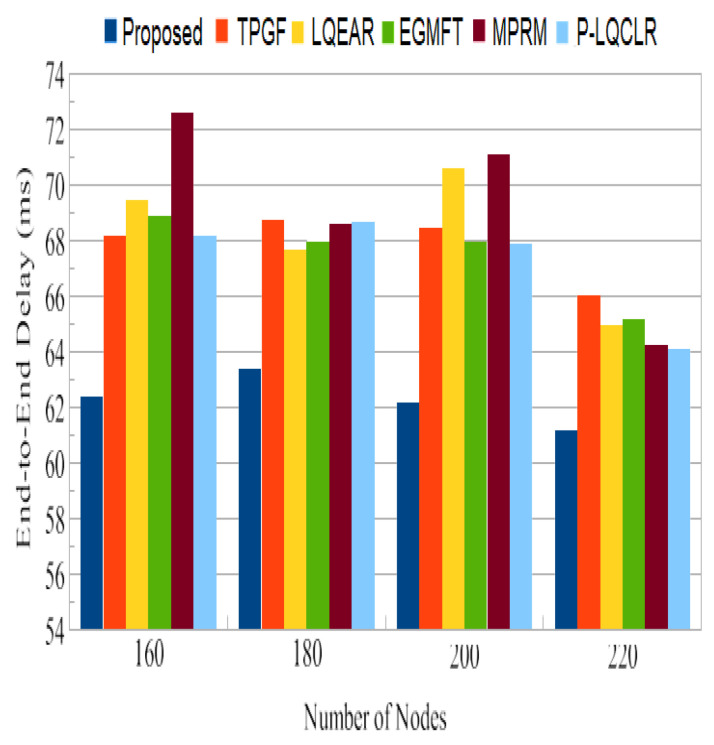
Standard end-to-end delay.

End-to-end delay: This measure shows how long it takes for an individual data packet to reach its destination after leaving the source node. The sink receives a total number of distinct data packets, and delays are averaged over all of those packets. Figure 12 demonstrates that the PROPOSED method distributes data packets more quickly than other protocols. In addition, as the WMSN applications are often delay-sensitive, our solution confirms that this method can offer the best delay in general. This result verifies that whenever paths are created with the expectation of the lowest inter-path interference effect, delay is reduced, which is often suitable for multimedia transmissions.

**Figure 13:**
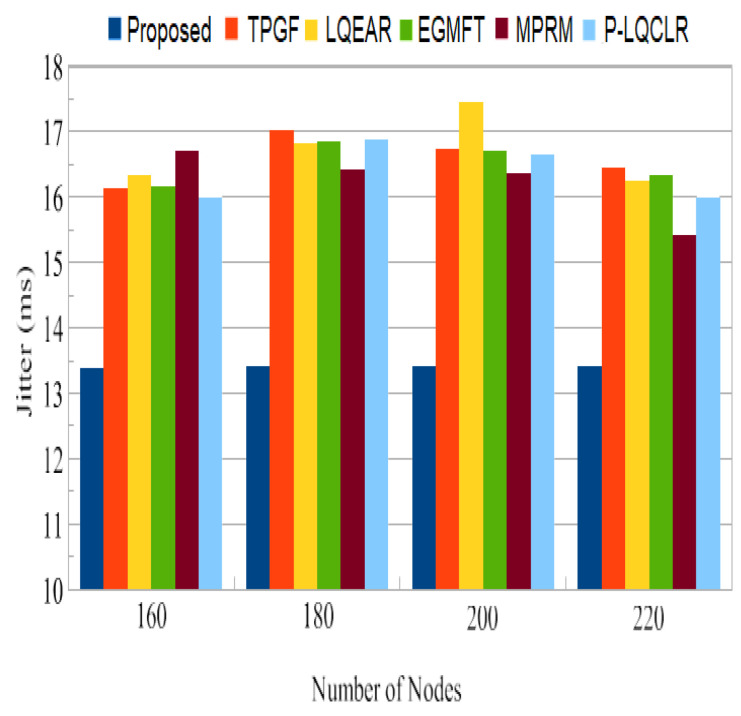
Average packet jitter.

Jitter: A fluctuation in the end-to-end delay of packets received at the sink is referred to as jitter. It occurs when certain packets travel from the source to the sink more slowly than others. Jitter should typically be minimized to achieve optimum perceived audio and video quality. From Figure 13, data packets can be seen to have little jitter, and the PROPOSED method achieved 16% to 20% less than other protocols. It can also be observed that jitter is quite high for other protocols; for this, which inter-path interference effect, the existence of high percentage of poor-quality links on active paths, and HNP at the sink node are to blame.

**Figure 14:**
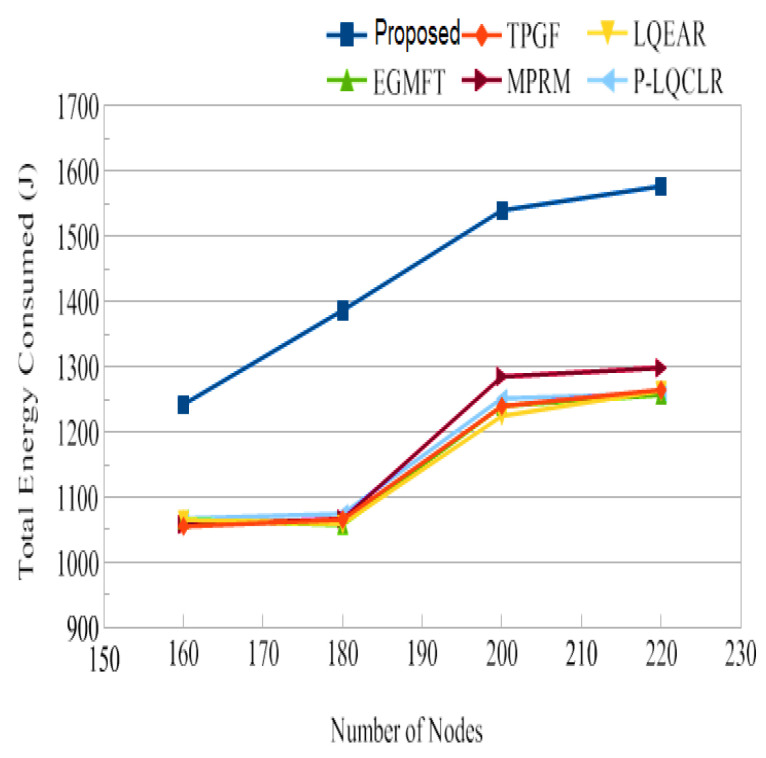
The average amount of energy used by the network.

**Figure 15:**
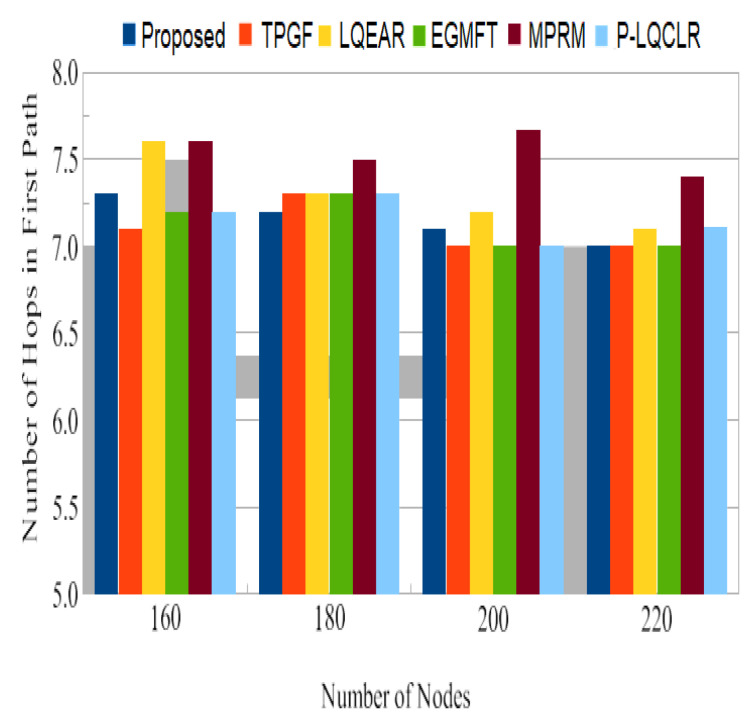
Hopping pattern in the initial path, on average.

**Figure 16:**
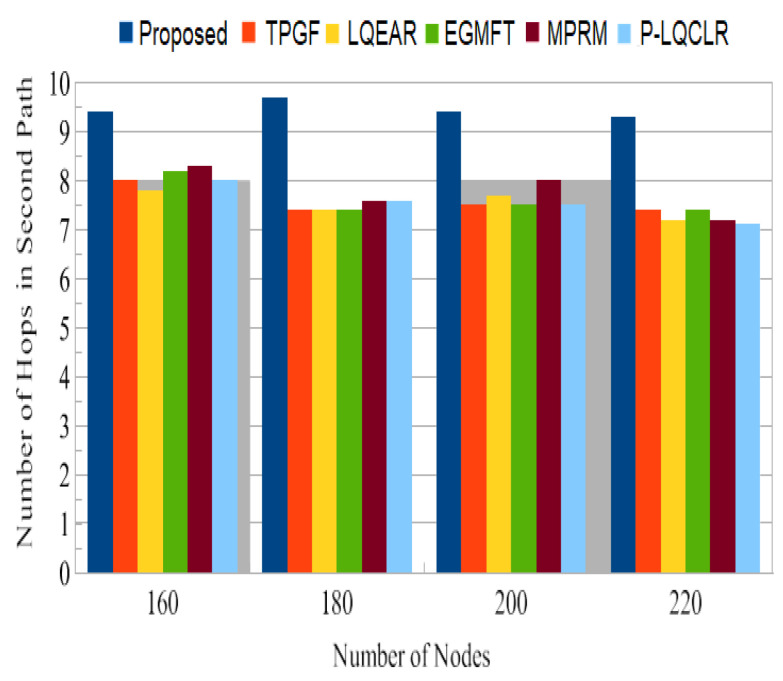
Hopping pattern in the second route, on average.

**Figure 17:**
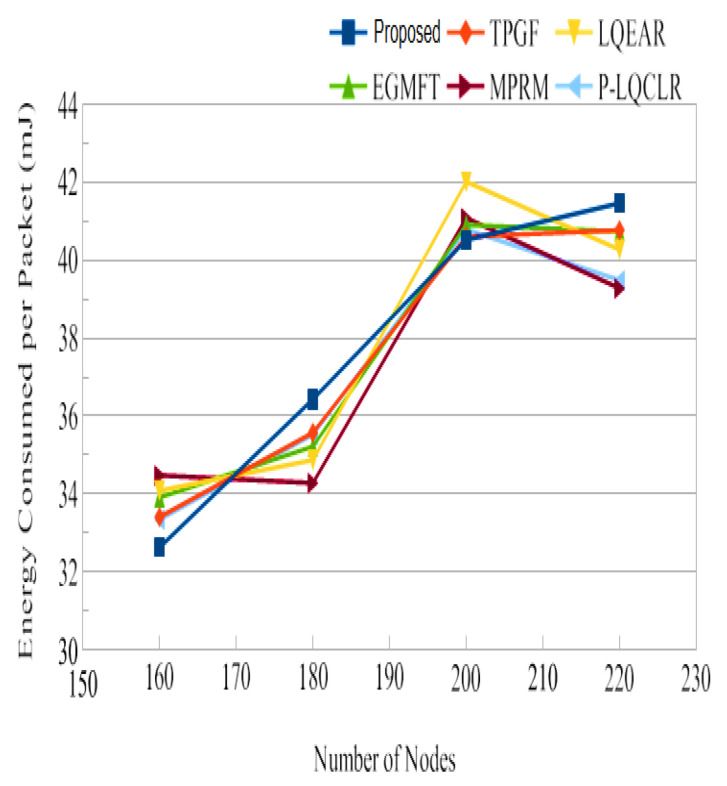
Energy use of an average packet.

Power consumption: Figure 14 presents the average total power consumption by the networks during the simulation time in joules. It has been found that TPGF and other protocols use 14% less energy than the PROPOSED method. This is primarily caused by the PROPOSED method’s delivery of additional data packets, transmission of small INFO packets during its neighbors’ discovery time, and the presence of extra hops on its derived second path. The first and second hops’ average numbers of paths are shown in Figure 15 and Figure 16. It has been noted that the initially discovered path has roughly the same number of hops as the TPGF protocol, while TPGF and other protocols have roughly two more hops than the PROPOSED method. Because we thought that there would be a minimal inter-path interference effect, we added these extra hops. The average amount of energy used per packet, as depicted in Figure 17, was about the same for all protocols regardless of the number of nodes. This was determined as the ratio of the network’s overall average energy usage to the sink’s overall average number of successfully delivered packages.

**Figure 18:**
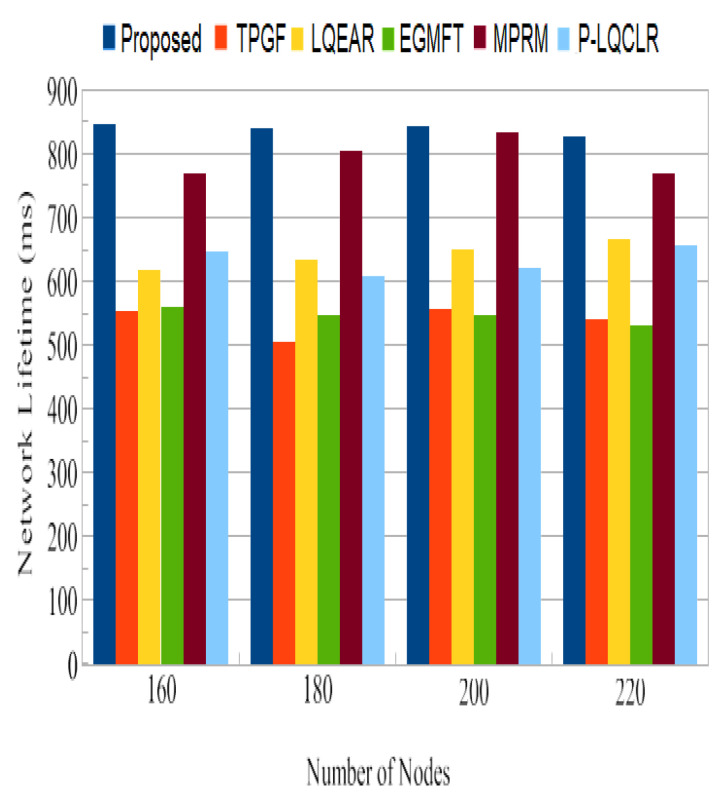
Network lifespan, on average.

**Figure 19:**
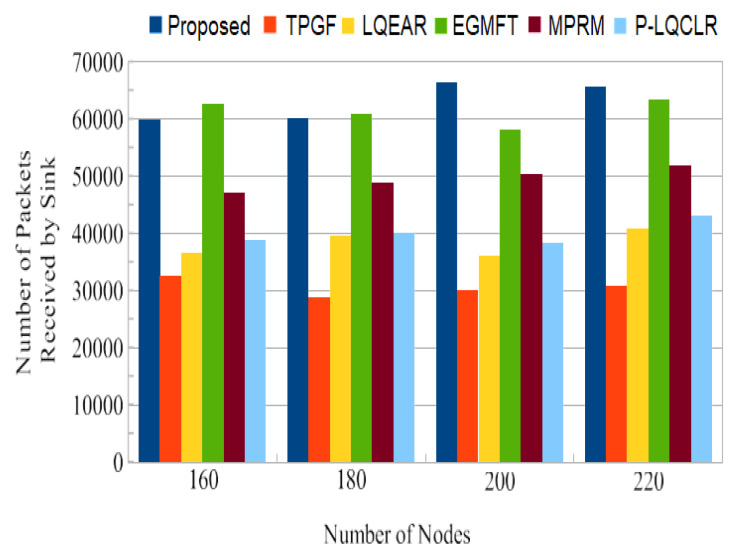
The number of packets the sink received during the second round of the 1200s simulation.

Network Lifespan: The length of time a network will last once it has been deployed is known as its lifespan. The simulation’s second round was run to determine precisely which initial node would exhaust all of its energy in order to determine the network’s lifetime. Figure 18 shows that TPGF and other protocols’ average network lifetimes were much less than those for PROPOSED. The energy of the relay node quickly depleted in the second round because the TPGF protocol chose the same relay nodes to establish pathways between the source and sink nodes. Figure 18 shows that one of the path’s intermediate nodes entirely used up all of its energy before becoming a DEAD node. The source nodes in the TPGF, LQEAR, MPRM, and P-LQCLR protocols are not aware of this circumstance, and continued sending data traffic in the direction of the sink node across this broken link. Also, subsequent data packets sent by the source node did not reach the sink node because of an intermediate DEAD node. Figure 19 shows that due to a broken connection on the path, TPGF, LQEAR, MPRM, and P-LQCLR delivered fewer data packets to the sink node. However, the PROPOSED protocol alerts source node when a path is likely to be broken and starts a soft transfer of data traffic across another active path, or investigates a new path. Figure 19 demonstrates that the PROPOSED protocol greatly outperformed TPGF, LQEAR, MPRM, and P-LQCLR in terms of data packets sent to the sink node thanks to an effective route management mechanism.

### 4.2. With Frame Retransmitting Scenario

This scenario enables resending of the frame in the IEEE 802.15.4 MAC sub-layer, which allows the base station nodes, and those of any intermediate route if necessary, to retransmit the data frame of a multimedia data transmission failure. In this instance, the simulation is conducted with a higher frame retransmission count of 1 and a network capacity of 160 nodes. It deletes the data frame of the MAC sub-layer after the nodes of the source and middle paths have made the greatest number of attempts to retransmit the frame. PDR, end-to-end delay, jitter, and energy usage are the network performance measures considered in the study. The simulation results for the 160-node network discussed in Section 4.2 are shown in this subsection with a retransmission count of 0 (i.e., no retransmission).

**Figure 20:**
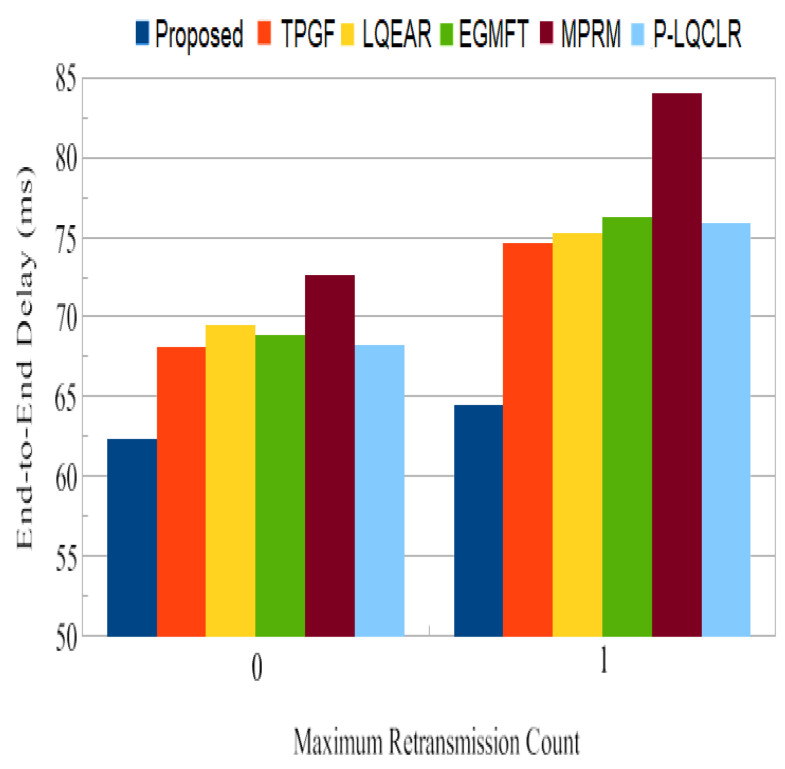
Standard end-to-end retransmission delays.

**Figure 21:**
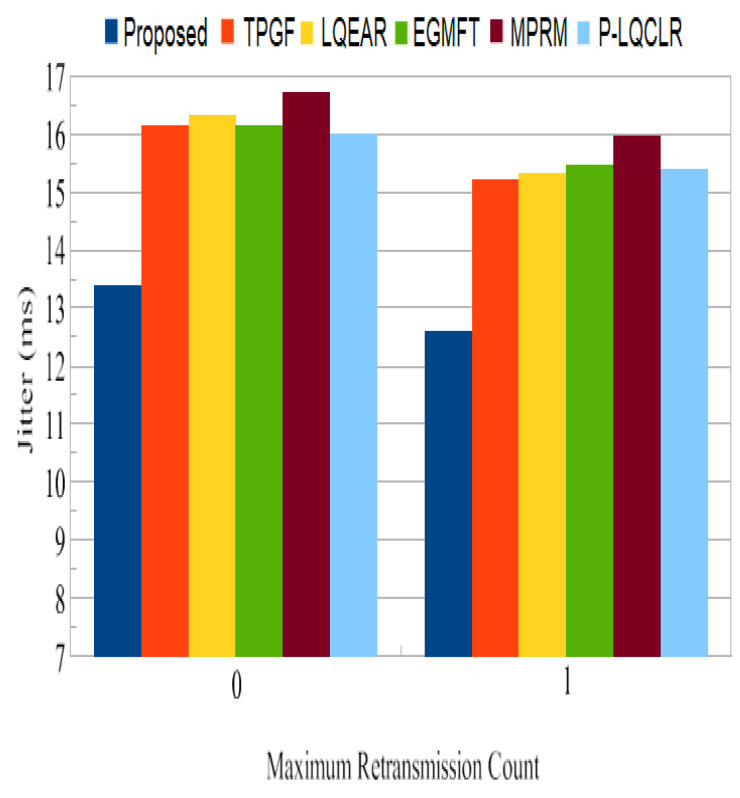
Average retransmission jitter.

PDR: Figure 20 presents the typical PDR with frame resend for a 160-node network. It can be observed that for PROPOSED, there was a more substantial upsurge in PDR for frame retransmission compared to other protocols. With retransmission, the average PDR for the PROPOSED protocol was nearly 99% against 76% to 81% for the remaining protocols. The results shown in Figure 21 support the findings in Figure 20 even more definitively. It has been noted that TPGF and other protocols have larger packet drops than the PROPOSED for 0 and 1 repetition count because the routing paths have more poor links than good links.

**Figure 22:**
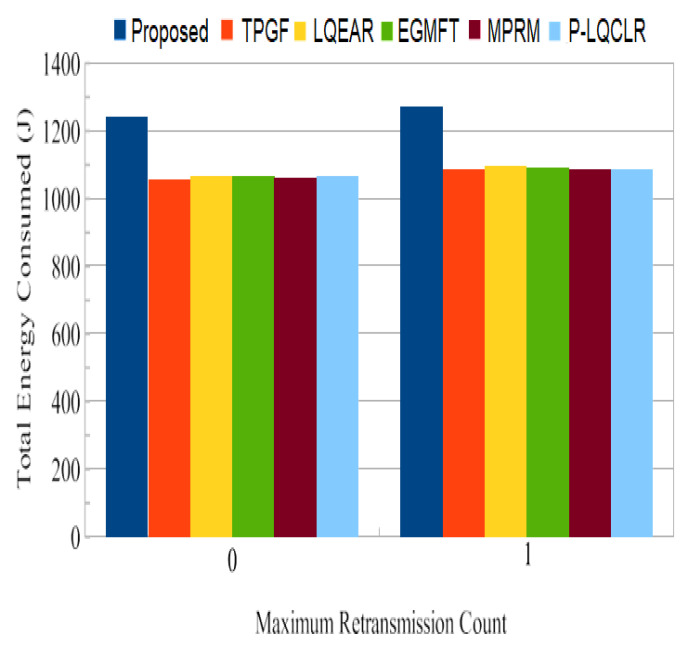
Retransmission-related average network energy consumption.

End-to-end delay: In addition to often dropping data frames, networks of poor quality also have greater delays as a result of data frame retransmissions. Figure 22 demonstrates that the PROPOSED protocol transfers packets from the source node to the sink node in a comparably shorter amount of time than the TPGF and other protocols with 0 and 1 frame retransmission counts. In addition, it can be observed that for other protocols, there was a more substantial upsurge in end-to-end delay for frame retransmission compared to PROPOSED. With a frame retransmission count equal to one, it was found that end-to-end delay was 16% higher for TPGF and 30% higher for MPRM when compared with PROPOSED.

**Figure 23:**
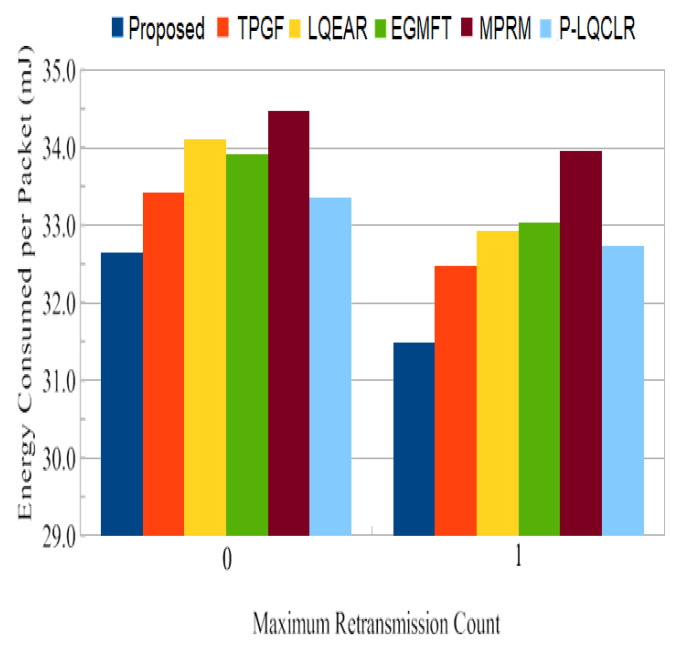
Average energy used for retransmission in a packet.

Jitter: Figure 23 shows that the PROPOSED protocol had lower jitter for data packets with 0 and 1 retransmissions than TPGF and other protocols.

Energy consumption: Figure 23 demonstrates the network’s total average energy consumption in joules for a 160-node network with retransmission counts of 0 and 1. It was found that PROPOSED uses more energy across the board than TPGF and other protocols. The same factors are at play as in the previous subsection. Nonetheless, Figure 23 shows that for frame retransmission with a network size of 160 nodes, the average power consumption per packet for PROPOSED was rather low when compared to other protocols. This is also shown in Table 3.

- Sensor node: the identifier for each sensor node in the network;
- Initial energy: the initial energy level assigned to each sensor node when the network is deployed, measured in joules (J);
- Energy consumption rate: the rate at which energy is consumed by each sensor node, measured in joules per second (J/s) or watts (W);
- Energy replenishment: the mechanism or source through which each sensor node replenishes its energy—this can include solar panels, battery recharge, wind turbines, or other methods;
- Energy threshold: the threshold energy level at which a sensor node becomes eligible for cluster head (CH) selection or rotation. This value determines the minimum energy required for a node to be considered for CH responsibilities.

## 5. Discussion

GSO is relatively simple to understand and implement compared to more complex optimization algorithms. It has fewer parameters to tune, making it more accessible for researchers and practitioners. The simplicity of GSO can be advantageous when time and resources are limited. GSO has also demonstrated favorable convergence properties in various optimization scenarios. Although convergence speed may vary depending on the problem’s complexity and the parameter settings, GSO generally converges to acceptable solutions within a reasonable number of iterations. This characteristic makes it suitable for optimization problems where quick convergence is desirable. The results presented in the previous section provide a comprehensive evaluation of the network performance characteristics for the PROPOSED protocol and other protocols in the context of multimedia data transmission. Here, we will discuss the findings and their implications.

PDR is a crucial metric for assessing the reliability of a routing system. The PROPOSED protocol achieved an average PDR close to 93%, which is significantly higher than the range of 73% to 81% observed for the other protocols. This indicates that the PROPOSED protocol is more effective in successfully delivering packets to the sink node. The higher PDR of PROPOSED can be attributed to its ability to identify paths with minimal adjacent path interference, reducing the chances of packet collisions and losses. Figure 11 shows that TPGF and other protocols experienced a disproportionately high number of data packet losses due to interference from neighboring nodes. This is attributed to the strong inter-path interference impact and the presence of heterogeneous high-power nodes (HNP) at the sink node. In contrast, the PROPOSED protocol demonstrates fewer packet losses in this scenario by selecting paths with less adjacent path interference. The results in Figure 12 indicate that the other protocols experienced 27% to 31% more packet losses due to poor link quality compared to the PROPOSED protocol. PROPOSED selected a higher percentage of good-quality links, as was estimated through the measure of triangle links’ quality, resulting in less packet loss. This highlights the effectiveness of PROPOSED in terms of mitigating the impact of poor link quality on packet delivery. The PROPOSED protocol demonstrated shorter end-to-end delay compared to other protocols, as shown in Figure 13. This indicates that PROPOSED can deliver data packets more quickly, making it suitable for delay-sensitive multimedia transmissions. The reduced delay can be attributed to the PROPOSED protocol’s ability to create paths with minimal inter-path interference effects.

Jitter, as shown in Figure 14, represents the fluctuation in the end-to-end delay of packets received at the sink. PROPOSED exhibited lower jitter compared to other protocols, indicating more consistent and predictable packet delivery. This is advantageous for maintaining high-quality audio and video transmissions. Figure 15 illustrates the average power consumption of the networks during the simulation. It can be observed that TPGF and other protocols consumed 14% less energy compared to PROPOSED. This can be attributed to PROPOSED delivering additional data packets and transmitting small INFO packets during neighbor discovery, as well as the presence of extra hops on the derived second path. However, Figure 18 shows that the average energy used per packet was similar for all protocols, regardless of the number of nodes. This implies that, on a per-packet basis, the energy efficiency is comparable among the protocols. The lifespan of a network after deployment is an important consideration. Figure 19 shows that the PROPOSED protocol achieved longer network lifespans compared to TPGF and other protocols. This is because the PROPOSED protocol effectively manages routes and avoids using relay nodes that quickly exhaust their energy. In contrast, other protocols may continue to send data traffic through broken links, leading to reduced network lifespans. Thereby, the evaluation and comparison of network performance characteristics highlight the superiority of the PROPOSED protocol in terms of PDR, end-to-end delay, jitter, and network lifespan. It demonstrated better performance in terms of successful packet delivery, reduced delay, lower jitter, and longer network lifespans compared to the other evaluated protocols. However, it should be noted that the PROPOSED protocol consumed more energy compared to other existing methods.

## 6. Conclusions

In this work, we created a distributed method for cluster head selection and a random algorithm for cluster formation to lower the expected energy consumption for high-traffic applications and next hop knowledge-based routing. According to the simulation results for the studied scenarios, the proposed distributed approach can cut down on energy use by up to 35% when compared to current practices. The proposed model improves energy efficiency and load balancing while minimizing throughput and latency. The main disadvantages of adjusting parameters are avoided when optimal parameters are selected using the GSO algorithm. The source of GSO was the animal’s search patterns. The framework was based on the producer–scrounger model; it was assumed that group members would look for opportunities to “find” (produce) or “join” (scrounger). It has also been discovered that selecting a reliable next hop and optimizing the routing protocol performance affect the link quality and interference intensity. The proposed priority queuing scheduling protocol incorporates a service differentiation model to ensure minimal queuing time, low jitter, and guaranteed reliability for very important traffic. As the overall performance of the network improves, advanced learning methods may be affiliated with ad hoc networks in the future, particularly in 5G networks.

## Figures and Tables

**Table 1:** K-NH Routing.

Cluster Head	Router Next Hop Count	CHk	Xi,k	Node	CHKs
1	2,3,5	3	(0.8 + 0.7 + 0.6)/3 = 0.7	3	5
2	3,4,6	3	(0.7 + 0.8 + 0.5)/3 = 0.67	2	4
3	5,7	2	(0.6 + 0.7)/2 = 0.65	2	7
4	7	1	0.7	1	7
5	8,15	2	(0.5 + 0.6)/2 = 0.55	2	15
6	1,13	2	(0.9 + 0.6)/2 = 0.75	2	13
7	8,9,10	3	(0.5 + 0.6 + 0.8)/3 = 0.63	2	9
8	9,15	2	(0.6 + 0.6)/2 = 0.6	2	15
9	15, base Station	2	(0.6 + 0.1)/2 = 0.8	2	starting point
10	starting point	1	starting point	2	starting point
11	10,12	2	(0.8 + 0.7)/2 = 0.75	1	12
12	14, base station	2	(0.7 + 0.1)/2 = 0.85	2	starting point
13	11,12,14	3	(0.9 + 0.7 + 0.7)/3 = 0.76	2	14
14	starting point	1	starting point	3	starting point
15	starting point	1	starting point	1	starting point

**Table 2:** Empirical parameters.

Parameters	Variant
Region for deployment simulations	200 m × 200 m
Number of nodes	[160–220]
Number of source nodes, location	1, (25, 100)
Number of sink nodes, location	1, (175, 100)
Radio propagation model	Two ray ground
MAC protocol: the physical and medium access control (MAC) layers for low-rate wireless personal area networks (LR-WPANs)	IEEE 802.15.4
Antenna	Omni antenna
Traffic type	64 kbps CBR
Transmit energy	0 dBm
Transmitter sensitivity	−97 dBm
Channel fidelity	2.4 GHz
Payload data size	100 bytes
Starting power	50 joules
Transmission power use	62.04 mw
Calculate power use	57.42 mw
simulated period	600 s
*α*, *β*, and *γ*	0.6, 0.2, and 0.2

**Table 3:** The energy status is depicted for four nodes.

Sensor Node	Initial Energy	Energy Consumption Rate	Energy Replenishment	Energy Threshold
Node 1	100 J	0.05 J/s	Solar Panel	30 J
Node 2	150 J	0.03 J/s	Battery Recharge	50 J
Node 3	120 J	0.04 J/s	Wind Turbine	40 J
Node 4	80 J	0.06 J/s	Battery Recharge	25 J

## Data Availability

The data presented in this study are available through email upon request to the corresponding author.
